# Application of Fully Convolutional Neural Networks in the Assessment of Cerebral White Matter Involvement in Primary Sjögren’s Syndrome

**DOI:** 10.1007/s12021-025-09762-1

**Published:** 2025-12-29

**Authors:** Michał Sobański, Miłosz Gajowczyk, Patryk Rygiel, Martyna Sobańska, Adrian Korbecki, Kamil Litwinowicz, Arkadiusz Kacała, Justyna Korbecka, Agata Zdanowicz-Ratajczyk, Edyta Dziadkowiak, Maciej Sebastian, Piotr Wiland, Grzegorz Trybek, Agata Sebastian, Joanna Bladowska

**Affiliations:** 1 Department of General Radiology, Interventional Radiology and Neuroradiology, Wroclaw Medical University, Wroclaw, Poland; 2 Wroclaw, Poland; 3 Mathematics of Imaging and AI, Department of Applied Mathematics, Technical Medical Centre, University of Twente, Enschede, The Netherlands; 4 Department of Maxillofacial Orthopaedics and Orthodontics, Wroclaw Medical University, Wroclaw, Poland; 5 Hetalox sp. z o.o, Wroclaw, Poland; 6 Clinical Department of Neurology, Medical University, Wroclaw, Poland; 7 Clinical Department of General, Minimally Invasive and Endocrine Surgery, Wroclaw Medical University, Wroclaw, Poland; 8 Clinical Department of Rheumatology and Internal Medicine, Wroclaw Medical University, Wroclaw, Poland; 9 Department of Oral Surgery, Pomeranian Medical University in Szczecin, Szczecin, Poland; 10 Faculty of Medicine, Wroclaw University of Science and Technology, Wroclaw, Poland; 11 Department of Radiology, Wroclaw 4th Military Clinical Hospital, Wroclaw, Poland

**Keywords:** DTI, Cerebral white matter, Primary sjögren's syndrome, PSS, Fully convolutional neural networks, Central nervous system

## Abstract

Central nervous system (CNS) involvement in primary Sjögren’s syndrome (pSS), although less frequent, can lead to serious complications. Our study aimed to assess white matter (WM) tract integrity, identify specific regions of disruption, quantify diffusion tensor imaging (DTI) metrics, and correlate these findings with rheumatologic factors. Thirty-three patients with pSS and twenty-six healthy subjects included in the control group, matched by gender and age were studied by performing brain DTI, which was reprocessed by the TractSeg algorithm based on fully convolutional neural networks (FCNN). The result was the segmentation of 72 main WM tracts, which were used to calculate quantitative values (fractional anisotropy – FA) of WM integrity. Finally, correlations of these values with rheumatological factors were made. Considering all WM tracts collectively, we observed significant differences between the study group and the control group. Numerous areas showed significant reductions in FA values, including novel observations involving all cerebellar peduncles and optic radiations. There were numerous significant correlations between altered FA values and particular clinical factors such as CRP level, haemoglobin level, presence of cryoglobulins and more. Our work unquestionably confirms and emphasises CNS involvement in pSS patients. Multiple impaired WM tracts correspond with symptoms associated with CNS, moreover, there were areas of impaired WM tracts previously not reported in DTI studies. Finally, multiple significant correlations were found with particular rheumatological factors, can indirectly indicate the influence of the severity of pSS on the integrity of WM tracts of CNS.

**Supplementary Information:**

The online version contains supplementary material available at 10.1007/s12021-025-09762-1.

## Introduction

Primary Sjögren’s syndrome (pSS) is a multifactorial disease involving genetic susceptibility and environmental factors (Helmick et al. 2008). Abnormal regulation is associated with pSS clinical manifestations (Li et al. 2013; Marketos 2019). pSS is heterogeneous, affecting multiple organs and systems, necessitating consideration of predominant lesions for effective therapy. Central nervous system (CNS) involvement in pSS, though less frequent, can lead to serious complications. Reported CNS involvement ranges from 2% to 60% (Moutsopoulos 1993; Andonopoulos 1990; Segal et al. 2008; Le Guern et al. 2009). Proposed mechanisms include small-vessel vasculitis and inflammatory processes (Alexander et al. 1981, 1994, 1988; Sakakibara et al. 2004; Tobón et al. 2012; Molina et al. 1985). Few reports describe CNS lesions, and their relationship with disease progression remains insufficiently explored (Segal et al. 2008; Tobón et al. 2012; Coates et al. 1999; Carvajal 2015; Andrianopoulou et al. 2020; Delalande et al. 2004; Margaretten 2017; Perzyńska-Mazan et al. 2018). This study aims to analyze brain changes in pSS patients at a near-cellular level using diffusion tensor imaging (DTI). DTI represents white matter (WM) tracts as a spatial map characterizing water diffusion in three dimensions, enabling assessment of tract organization and diffusion along axonal membranes. Many developmental processes, as well as brain ageing and pathological processes occurring in the CNS affect the microstructural composition and architecture of WM. This results in impaired water diffusion along WM fibres which is referred to as impaired integrity of WM tracts. Our work focuses on the analysis of fractional anisotropy (FA) values, which, alongside mean diffusivity (MD), are most commonly used to determine the microstructure of WM tracts. The use of FA, bypassing MD analysis, is related to the fact that the calculation of MD values carries some limitations and simplifications. The FA value determines the fraction of diffusion intensity that can be attributed to anisotropic diffusion. FA values vary between 0 (isotropic diffusion) and 1 (infinite anisotropy) (Westin 2002; Basser 1996; Le 2001; Ranzenberger 2023).

### Objectives of the Study

This study aims to assess the value of an advanced magnetic resonance imaging (MRI) technique such as DTI in imaging of WM disruption using:

1. Demonstrating impaired cerebral white matter integrity measured by DTI parameters.
2. Identifying specific areas with impaired integrity of WM tracts which means decreased FA values.
3. Determining correlations between DTI-derived quantitative measures and clinical or laboratory manifestations of pSS.
4. Distinguishing the AI algorithms in the processing of DTI.

### Materials

Thirty-three patients with pSS and twenty-six healthy subjects included in the control group, matched by gender and age were studied by performing brain MRI examinations. Consent to conduct the study has been given by the bioethics committee working by Wroclaw Medical University – opinion number KB − 578/2020. All subjects gave written informed consent after receiving a full explanation of the study procedures. Participation was voluntary, and participants were free to decline or withdraw at any stage without affecting their clinical care. The study group consisted of patients who met the 2016 ACR/EULAR classification criteria for pSS (Shiboski et al. 2017). Further inclusion criteria were: absence of symptoms of central or peripheral nervous system involvement, age ≥ 18 years, and written consent. The exclusion criteria consisted of the coexistence of other connective tissue diseases, infectious diseases (including viral infection within 3 months prior to MRI examination, also COVID-19), and any neurologic and psychiatric diseases. All patients were examined by a neurologist to exclude any neurological pathology. The clinical data obtained from the study group were as follows: age at MRI examination, sex, duration of disease, age at diagnosis of pSS, presence of eye dryness, joint involvement, presence of skin symptoms, presence of pulmonary symptoms and other rheumatological factors. The ESSDAI (EULAR Sjögren’s syndrome disease activity index) questionnaire was used to quantify the severity of disease activity (12 domains, no disease activity/low/moderate or high disease activity) and the ESSPRI (EULAR Sjögren’s syndrome patient reported index) was used to assess the severity of dryness symptoms (0-no symptoms; 10 points the patient’s maximum perceived severity of dryness), pain, and fatigue (Seror et al. 2011). Laboratory tests were conducted such as blood morphology, antinuclear antibodies (ANA), anti-SSA and anti-SSB antibodies, rheumatoid factor (RF), cryoglobulins, and γ-globulins. Additional data, including coexisting morbidities (e.g., hypertension, hypothyroidism, Hashimoto disease) and prescribed medications (e.g., hydroxychloroquine, vitamin D3, steroids), were also collected.

## Methods

### MRI examination

The study and control groups underwent brain MRI on a 3 T Philips Ingenia scanner using a 32-channel head and spine coil. The study protocol consisted of axial T2-weighted images, susceptibility-weighted images, T1-weighted 3D BRAVO, and 3D FLAIR as well as diffusion tensor imaging (DTI). The DTI sequence was performed using single-shot turbo spin echo echo planar imaging sequences in 12 coding directions, b = 700, time of elongation = 59ms, time of relaxation = 3000ms, flip angle = 90ᵒ, 140 × 140 matrix, voxel size = 2.5$\:m{m}^{2}$, slice thicknes = 2.5 mm, acquisition in sagittal projection, field of view = 349 mm.

### Description of methodology

**Fig. 1:**
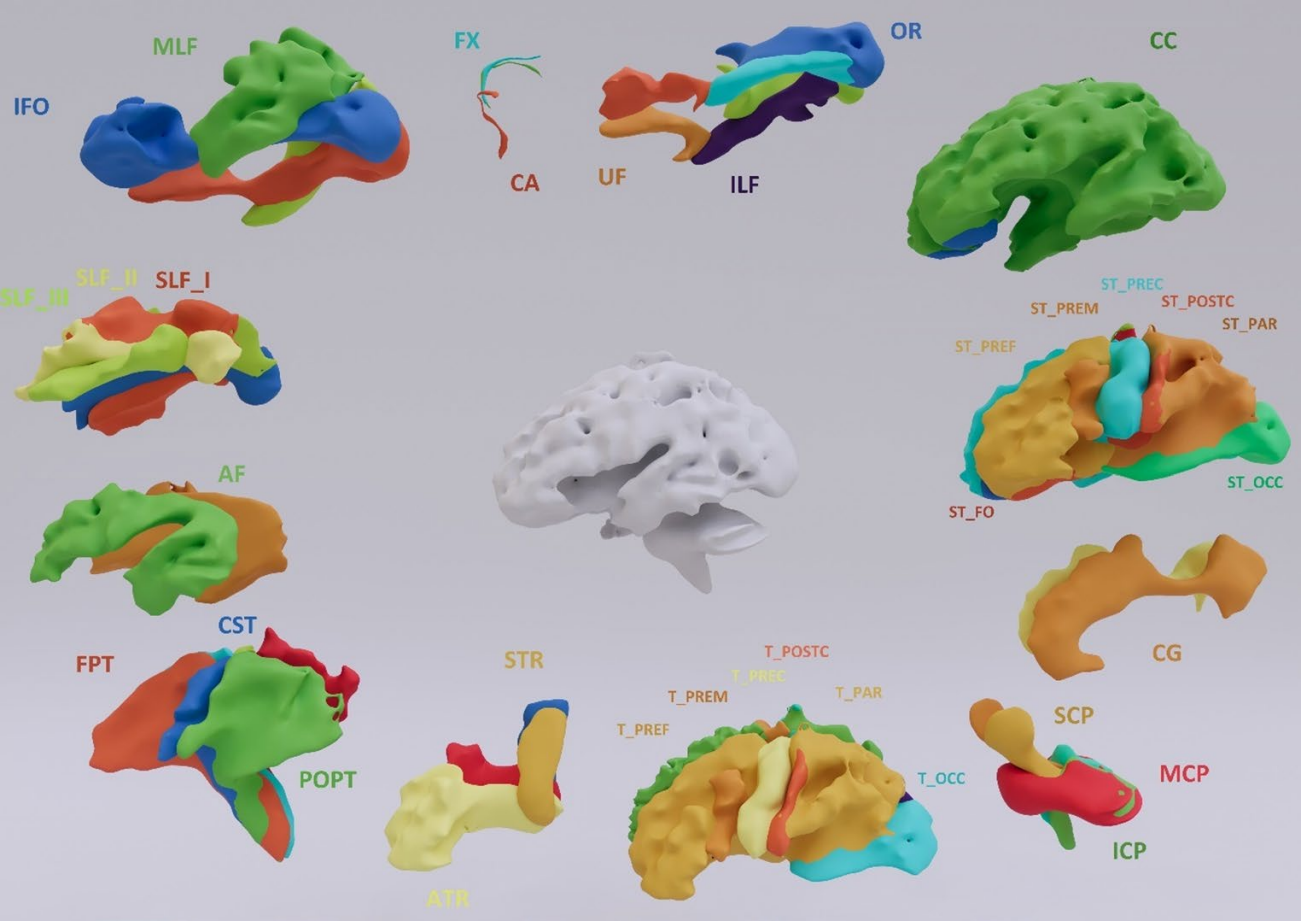
Overview of all 72 WM tracts of the brain (for tracts that occur in both the left and right cerebral hemispheres, only the right-tract is shown in the figure). **WM tracts abbreviations** arcuate fascicle- AF; anterior thalamic radiation – ATR; commissure anterior – CA; corpus callosum – CC (rostrum– CC1, genu – CC2, rostral body – CC3, anterior body – CC4, posterior body – CC5, isthmus – CC 6, splenium - CC7), anterior thalamic radiation – ATR; cingulum – CG; corticospinal tract – CST; middle longitudinal fascicle – MLF; fronto-pontine tract – FPT; fornix – FX; inferior cerebellar peduncle – ICP; inferior occipito-frontal fascicle – IFOF; inferior longitudinal fascicle – ILF;. parieto-occipital pontine tract – POPT; superior cerebellar peduncle – SCP; superior longitudinal fascicle I – SLF I; superior longitudinal fascicle I – SLF II; superior longitudinal fascicle III – SLF III; superior thalamic radiation – STR; uncinate fascicle – UF; thalamo-prefrontal tract – T_PREF; thalamo-premotor tract – T_PREM; thalamo-precentral tract – T_PREC; thalamo-postcentral tract – T_POSTC; thalamo-parietal tract – T_PAR; thalamo-occipital tract – T_OCC; ang. striato-fronto-orbital tract– ST_FO; striato-prefrontal tract – ST_PREF; striato-premotor tract – ST_PREM; striato-precentral tract – ST_PREC; striato-postcentral tract – ST_POSTC; striato-parietal tract – ST_PAR; striato-occipital tract – ST_OCC

In our study we implemented the TractSeg algorithm introduced by J. Wasserthal in 2018 which is based on fully convolutional neural networks (FCNN) (Wasserthal et al. 2018). The final output consists of binary segmentations of the 72 principal WM tracts (Fig. 1). Detailed description of the methodology can be found in supplementary data 1. After the segmentation of 72 WM tracts, the mean FA value was calculated based on three eigenvalues obtained from the covariance matrix of tensor diffusion (Basser 1996). All steps required to calculate FA values are included in supplementary data 2.

### Statistical Analysis

**Fig. 2:**
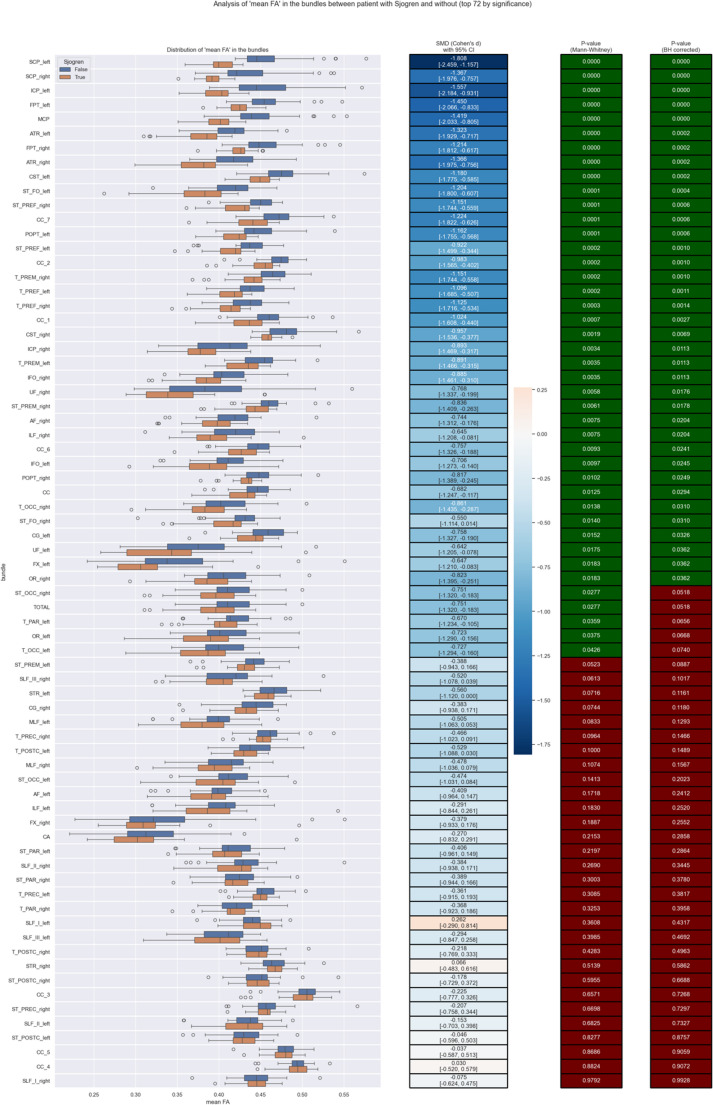
Comparison of mean fractional anisotropy (FA) values between patients with primary Sjögren’s syndrome and healthy controls across 72 white-matter bundles. Left panel: boxplots showing the distribution of mean FA in each tract for the Sjögren group (orange) and controls (blue). Middle panel: standardized mean differences (SMD; Cohen’s d) with corresponding 95% confidence intervals, illustrating the magnitude and direction of group differences (negative values indicate lower FA in patients). Right panels: p-values derived from the Mann–Whitney U tests (green) and Benjamini–Hochberg FDR-corrected p-values (green to red gradient), ordered from the most to the least significant tracts. The figure demonstrates widespread FA reduction in the Sjögren group, with the majority of tracts remaining significant after FDR correction

Differences in mean fractional anisotropy (FA) between patients with primary Sjögren’s syndrome and healthy controls were evaluated for each of the 72 white-matter tracts. Because FA values were not normally distributed, group comparisons were performed using the non-parametric Mann–Whitney U test. For each tract, the effect size was quantified using the standardized mean difference (SMD, Cohen’s *d*) together with its 95% confidence interval, calculated using pooled standard deviations. To account for multiple comparisons across all tracts, p-values from the Mann–Whitney U tests were adjusted using the Benjamini–Hochberg false discovery rate (FDR) method. Tracts were ranked by statistical significance (FDR-corrected *p*-values), and all results were visualized using combined boxplots and effect-size heatmaps (Fig. 2). Statistical significance was set at *p* < 0.05 after correction.

**Fig. 3:**
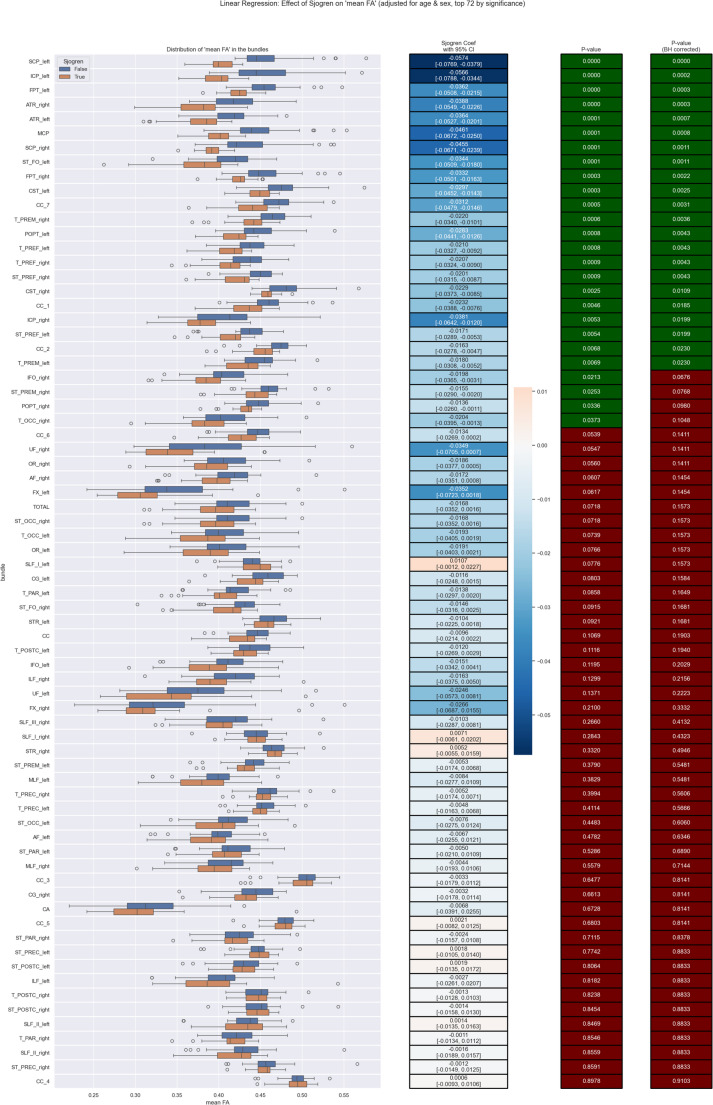
Linear regression analysis demonstrating the effect of primary Sjögren’s syndrome on mean fractional anisotropy (FA) across 72 white-matter bundles, adjusted for age and sex. Left panel: boxplots illustrating unadjusted distributions of mean FA in patients with Sjögren’s syndrome (orange) and healthy controls (blue). Middle panel: regression coefficients for the Sjögren predictor with 95% confidence intervals, representing the adjusted effect of the disease on FA (negative coefficients indicate lower FA in the Sjögren group). Right panels: corresponding p-values and Benjamini–Hochberg FDR-corrected p-values, sorted by significance. A widespread pattern of negative coefficients is observed, with several projection and association pathways showing significant FA reduction after covariate adjustment

To assess the independent effect of primary Sjögren’s syndrome on white-matter microstructure, a linear regression model was fitted for each of the 72 white-matter bundles, with mean fractional anisotropy (FA) as the dependent variable. Sjögren status (patient vs. control) was entered as the main predictor, and all models were adjusted for age and sex. For each tract, regression coefficients with corresponding 95% confidence intervals were obtained to quantify the magnitude and direction of the association. Statistical significance of the Sjögren coefficient was evaluated using two-sided *p*-values. To control the false discovery rate across multiple comparisons, *p*-values were adjusted using the Benjamini–Hochberg procedure. Results were ranked according to FDR-corrected significance and visualized using combined boxplots and regression-coefficient heatmaps (Fig. 3).

Group comparisons between patients with pSS and healthy controls were performed using the Mann–Whitney U test due to non-normal distribution of diffusion metrics. Associations between clinical variables and tract–specific mean FA values were assessed using Pearson correlation coefficients. To account for the large number of comparisons across 72 white-matter tracts and multiple clinical parameters, *p*-values were adjusted using the Benjamini–Hochberg procedure, controlling the false discovery rate at q < 0.05. Several correlations showed moderate effect sizes in the uncorrected analysis but did not remain statistically significant after correction for multiple testing. These findings should therefore be interpreted cautiously and warrant replication in larger cohorts to confirm potential clinical–microstructural relationships.

## Results

Analysis of mean FA revealed striking and widespread disruption of white-matter microstructure in primary Sjögren’s syndrome (Fig. 2). The strongest effects were found in cerebellar peduncles, anterior thalamic radiations, fronto-pontine tracts, and corticospinal tracts, all showing large standardized mean differences (Cohen’s *d* raging from − 1.5 to − 2.5) and highly significant Mann–Whitney U test results. Numerous association tracts, including the cingulum, superior longitudinal fasciculus, and inferior fronto-occipital fasciculus, also showed significant reductions. More than half of the 72 examined bundles remained significant after Benjamini–Hochberg correction. These findings indicate a diffuse pattern of white-matter microstructural disruption in Sjögren’s syndrome.

Patients with primary Sjögren’s syndrome demonstrated a consistent pattern of reduced mean FA across multiple white-matter bundles, even after adjusting for age and sex (Fig. 3). After adjusting for age and sex, the direction of the disease effect remained unchanged, consistently demonstrating lower FA in patients with Sjögren’s syndrome. Although covariate adjustment slightly reduced effect sizes and reduced the number of marginally significant findings, the core set of highly affected tracts remained significant, confirming that the observed alterations are robust and not attributable to demographic confounding. The stability of these results supports a true disease-related disruption of white-matter microstructure rather than an age- or sex-driven effect.

A comparison of unadjusted and covariate-adjusted analyses demonstrated high concordance in the pattern and direction of FA abnormalities in primary Sjögren’s syndrome. Both approaches revealed widespread FA reduction, with the strongest effects localized to the cerebellar peduncles, anterior thalamic radiations, fronto-pontine tracts, corticospinal tracts, and major association pathways. Adjustment for age and sex resulted in slightly attenuated effect magnitudes and fewer FDR-significant tracts; however, the core set of strongly affected bundles remained significant in both analyses. This consistency indicates that the observed FA alterations are robust and not attributable to demographic confounding, reinforcing the presence of widespread white-matter microstructural disruption in Sjögren’s syndrome.

**Fig. 4:**
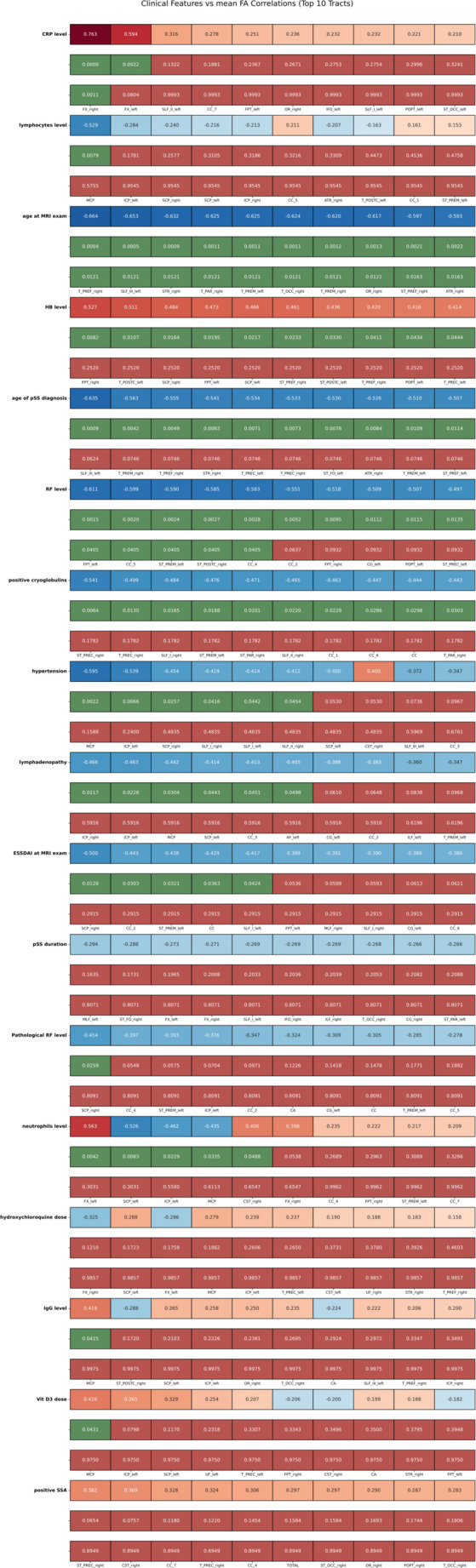
Correlation heatmap demonstrating relationships between clinical characteristics and mean fractional anisotropy across the 10 white-matter tracts showing the strongest associations. For each clinical–tract pair, the top line displays the Pearson correlation coefficient (r), the middle line the corresponding uncorrected p-value, and the bottom line the Benjamini–Hochberg corrected p-value. This layout enables simultaneous assessment of effect size, unadjusted significance, and significance after multiple-testing correction

Across the analysed cohort, several clinical variables demonstrated meaningful associations with tract-specific mean FA values (Fig. 4). The strongest correlations were observed for markers of current inflammation and disease activity. The CRP level showed the most pronounced effect, with robust positive correlation in right fornix (r up to 0.76), which remained statistically significant after Benjamini–Hochberg correction, indicating stable association.

Indicators of hematological status–hemoglobin level and lymphocyte count–showed moderate correlations (*r* ≈ 0.42–0.53 and *r* ≈ − 0.21 to − 0.53, respectively), but none of these survived BH correction, suggesting weaker or sample-size–limited effects. The RF level exhibited several significant negative correlations with FA (up to *r* = − 0.61), and a subset remained significant after BH correction, indicating a relatively robust relationship between RF level and microstructural involvement. In contrast, pathological RF positivity demonstrated only one nominally significant association at the Pearson level, which did not persist after BH adjustment, suggesting a modest or tract-restricted effect. The presence of cryoglobulins showed consistent negative correlations (*r* ≈ − 0.44 to − 0.54), significant before correction but none remaining significant after BH, reflecting a trend toward reduced FA in cryoglobulin-positive patients. Comorbid clinical conditions showed smaller effects. Hypertension and lymphadenopathy were associated with lower FA across several tracts, with Pearson correlations reaching significance in selected regions, but none survived BH correction, indicating only subtle microstructural impacts. Measures of systemic disease burden, including ESSDAI score, showed the expected negative associations with FA (*r* ≈ − 0.39 to − 0.50), though again these did not remain significant following BH correction. For treatment-related variables (hydroxychloroquine dose, vitamin D dose) and immunologic markers (IgG, positive SSA), correlations were weak to modest and entirely non-significant after correction. Overall, only CRP and selected RF-related correlations demonstrated BH-resistant associations, while most other clinical parameters showed directionally meaningful but statistically attenuated effects, suggesting subtle microstructural changes detectable only at uncorrected significance levels.

## Discussion

The pathomechanism of CNS changes in pSS remains unclear, with studies proposing a vasculogenic process that may lead to reduced perfusion as a primary substrate (Andrianopoulou et al. 2020; Moreira et al. 2015; Segal et al. 2010). Post-mortem studies indicate small-vessel disease as an important factor, (Alexander 1993) while SPECT and PET studies show hypoperfusion and reduced glucose metabolism (Huang et al. 2003; Kao et al. 1998). DTI studies revealed disrupted WM tract integrity, potentially related to Wallerian degeneration, inflammation-induced hypoperfusion, or cytokine dysregulation affecting the vascular endothelium (Segal et al. 2008, 2010; Tobón et al. 2012; Andrianopoulou et al. 2020; Moreira et al. 2015; Massara et al. 2010; Morreale 2014; Tzarouchi et al. 2014). Neurological symptoms attributable to CNS involvement in pSS are estimated to occur in 2% to 60% of patients, but lack of a defined neurological form and early disorders like mild cognitive impairment pose diagnostic challenges. Non-specific symptoms include fatigue, impaired memory, and concentration, while specific CNS symptoms encompass various conditions (Tobón et al. 2012; Andrianopoulou et al. 2020; Moreira et al. 2015; Massara et al. 2010; Morreale 2014). Conventional MRI findings, such as WMHs, are inconclusive (Andrianopoulou et al. 2020; Moreira et al. 2015; Segal et al. 2010). Advanced techniques, like DTI, offer insights into cellular-level CNS changes, revealing abnormalities in FA and MD values indicative of disrupted WM tract integrity.

### Key Findings

In our study patients with pSS exhibited a significant reduction in FA value in several WM tracts, including a wide range of thalamocortical tracts. These changes may stem from decreased myelination, reduced axonal density, and disrupted fibre organization in WM tracts (Mamah et al. 2010). The altered structure in these fibres can impact transmission between the thalamus and the prefrontal and orbitofrontal cortex, affecting emotional processes and leading to memory disturbances (Mamah et al. 2010; Fenlon et al. 2021). Memory impairment, even without emotional disturbances, may occur. Tracts projecting to the prefrontal and orbitofrontal cortex play a role in higher brain functions, and disturbances can affect cognitive processes, memory, and habituation. Pathologies in this area during the acute phase may result in memory processing loss and message acquisition (Fenlon et al. 2021).

Significant decreases in FA values were observed in specific regions of corpus callosum (CC), with reductions in FA values in the anterior part (rostrum – CC1 and genu – CC2) and posterior part of CC (isthmus – CC6 and splenium – CC7). Notably, the reduction in FA values in the anterior part was significantly more pronounced than in the age-matched control group, suggesting factors beyond age-proportional changes (Fabri 2014). The rostrum and genu are crucial for prefrontal cortex connectivity, while the splenium connects the occipital and partially temporal cortex. Bilateral connectivity disruption in the anterior corpus callosum may impact cognitive and emotional functions. Abnormalities in this area are found in dyslexic patients, those with Down syndrome, and various types of dementia, including Alzheimer’s Disease and frontotemporal dementia (Fabri 2014). FA reduction in these CC regions may serve as a predictor of neuropsychiatric changes in pSS. The splenium’s involvement in visual processing and motor-sensory transmission emphasizes the potential overall negative impact of pathology occurring in both the anterior and posterior parts of the CC (Fabri 2014; Zarei et al. 2006).

We found a significant reduction of FA value in all cerebellar peduncles in pSS, to the best of our knowledge, such findings have not been previously reported. Cerebellar damage is typically associated with locomotor disturbances, hemispheric syndrome and movement breakdown during complex activities (Moryś 2021). Our study revealed significant reduction of FA values in both fronto–pontine tracts, suggesting predictive pathological alterations in fronto–pontine–cerebellar tracts. Disruption in these tracts may impair procedural memory processes, affecting the learning and execution of complex skills (Janusz 2021; Jissendi et al. 2008). Monitoring cerebellar WM changes can aid early disease detection, diagnose CNS symptoms in pSS, and indicate treatment effectiveness. Notably, DTI studies on cerebellar WM in multiple sclerosis (MS) patients without typical lesions on conventional MRI showed a correlation between disability and disease duration, reinforcing the potential benefit of early detection in MS (Deppe et al. 2016).

Furthermore, we observed a significant reduction in FA values in both optic radiations, a previously unreported observation. Reduction of FA values lost significance after adjusting for age and sex, which can indicate that analyzed group of patients was too small or observed group difference is explained by demographic variability rather than disease status alone. Similar reductions in FA value have been found in autoimmune diseases like neuromyelitis optica spectrum disorders (NMOSD), (Jeantroux et al. 2012; Pache et al. 2016)which has been suggested to fall within the spectrum of CNS involvement in pSS (Morreale 2014). The reduction in FA value is likely due to secondary Wallerian degeneration, possibly arising from alterations in anatomical regions typically affected in NMOSD, particularly optic nerve involvement (Jeantroux et al. 2012).

An important finding was the significant reduction of FA value in both uncinate fasciculi, impacting emotional processes. The reduction of FA values lost significance after adjusting for sex and age, which can be explained by similar factos as mentioned above. Such alteration in uncinate faciculi was also reported in various psychiatric diseases and conditions with behavioural disorders. Studies focused on frontotemporal dementia (FTD), semantic dementia (SD), and progressive nonfluent aphasia (PNFA) showed disruption in both uncinate fasciculi in the behavioural variant of FTD, correlating with the degree of behavioural disorders. Atrophy of the grey matter connecting the uncinate fasciculus was also observed (Hornberger et al. 2011; Mahoney 2012). Another study on patients with post-traumatic brain disorders revealed abnormalities in the microstructure of the left uncinate fasciculus and left cingulate gyrus, affecting symptoms such as apathy and depression (Zappalà et al. 2012). In children with traumatic brain injury, there was a significant correlation between FA values with impaired emotion control (Johnson et al. 2011). Nonetheless, psychiatric disorders are more common in patients with pSS, contributing to difficulties in emotional recognition, somatosensory sensations, and impaired cognitive function.

We also observed a statistically significant reduction of FA values in both pyramidal tracts (cortico-spinal tracts – CST) most likely resulting in neurological deficits. Abnormalities in CST microstructure and concomitant striate-cortical tracts may result in Parkinsonian syndromes in patients with pSS. Most of which are atypical Parkinsonian syndromes with symmetric, rigid, akinetic tremors and balance disturbances (Tobón et al. 2012; Moreira et al. 2015). It is important to bear in mind that Parkinson’s syndromes in the course of pSS are poorly understood, so further research in this area is needed.

### Comparison with current literature

Although several studies have examined tract-based DTI alterations in MCI and Alzheimer’s disease, (Zhuang et al. 2012; Teipel et al. 2011) the present work is the first to apply a fully automated FCNN-based pipeline (TractSeg) for whole-brain, tract-specific analysis across 72 pathways. Earlier ROI-based approaches suffer from operator dependence and limited spatial sampling, whereas TBSS projects diffusion values onto a WM skeleton, reducing anatomical specificity and increasing sensitivity to registration errors. Traditional tractography pipelines similarly require multiple manual or semi-manual steps, introducing variability and limiting reproducibility (Saha 2020; Tchetchenian et al. 2023).

TractSeg addresses these limitations by directly segmenting anatomically defined bundles from FOD peaks without classical tractography, enabling fast, robust, and operator-independent segmentation with high anatomical precision. Critically, TractSeg has demonstrated strong performance even on down-sampled, clinical-quality and artefact-affected data, as well as across scanners and pathological conditions (Wasserthal et al. 2018, 2019). Subsequent studies confirmed its status as a state-of-the-art method, with only marginal gains from architectures specifically optimized for lower-quality datasets (Saha 2020; Tchetchenian et al. 2023). These characteristics—together with publicly available code and pretrained weights—make TractSeg a highly reproducible and scalable tool for clinical and multi-centre DTI studies such as pSS. DTI studies in primary Sjögren’s syndrome (pSS) consistently indicate microstructural white-matter (WM) involvement, although methods and spatial coverage vary substantially across reports. Early work by Segal B.M. et al. demonstrated reduced FA and increased MD in a single frontal lobe voxel, with the lowest FA observed in pSS patients with CNS symptoms (Segal et al. 2010). While their highly restricted ROI limits comparability with tract-level analyses, the sampled region corresponds to terminal projections of several tracts that also showed FA reductions in our study, including the anterior thalamic radiations, striatal–prefrontal and thalamic–prefrontal pathways, the inferior fronto-occipital fasciculus, the uncinate fasciculus, and the right inferior longitudinal fasciculus.

Tzarouchi L.C. et al. applied slice-wise voxelwise mapping and demonstrated dispersed clusters of reduced FA involving the corticospinal tracts, superior longitudinal fasciculus, anterior thalamic radiations, inferior fronto-occipital fasciculus, uncinate fasciculus, and inferior longitudinal fasciculus (Tzarouchi et al. 2014). These partially overlap with our findings, particularly within the pyramidal tracts, inferior fronto-occipital fasciculi, uncinate fasciculi, and the right inferior longitudinal fascicle. Similarly, Andrianopoulou A. et al. used voxelwise and cluster-based approaches to show differences in FA/MD between depressive and non-depressive pSS patients, and between non-depressive pSS and controls, with abnormalities across the anterior thalamic radiations, pyramidal tracts, cingulum, forceps minor/major, and both longitudinal fasciculi (Andrianopoulou et al. 2020) - closely mirroring the widespread pattern we identified.

More recently Zhang et al. (2022) examined pSS using whole-brain structural connectivity (SC) analysis based on graph-theoretical link-based comparisons. They found decreased SC predominantly within the frontoparietal network, alongside additional alterations in temporal, occipital and insular connections, and one instance of increased caudate–cingulate SC suggestive of compensatory reorganization. Importantly, reduced SC between the middle temporal and middle occipital gyri correlated with WMH burden (Zhang et al. 2022). Although this approach differs from tract-specific FA quantification, their demonstration of distributed frontoparietal and temporo-occipital abnormalities aligns strongly with our tract-level findings and supports the view that pSS produces broad, network-level disturbances in WM integrity.

### Clinical Use

A statistically significant strong positive correlation (*r* = 0.76 for the right side, *r*= 0.60 for the left side) between CRP levels and FA values in the bilateral fornix was observed. These results indicate that higher CRP levels are strongly associated with increased FA in the fornix, and while both sides showed significant correlations, only the right fornix remained statistically significant after correction for multiple testing, suggesting that this association is particularly robust on the right side. CRP is produced in response to pro-inflammatory interleukin-6 (IL-6) (Black et al. 2004). However, in systemic connective tissue diseases like pSS, CRP may not reliably indicate inflammation, as demonstrated by low levels despite elevated IL-6 (Bianchi et al. 2022; Witas et al. 2020). Changes in FA values were more pronounced in patients with lower CRP concentrations in our study, challenging conventional expectations. CRP, involved in immune system regulation, can bind ligands and mediate interactions with Fc receptors, contributing to phagocytosis (Lu et al. 2008, 2018; Mortensen et al. 1976). The observed variations in CRP response may be associated with polymorphisms and down-regulation in certain diseases, like systemic lupus erythematosus (SLE) (Enocsson et al. 2014, 2021; Sebastian et al. 2022). However, CRP is considered an unreliable marker for monitoring disease activity in pSS, and further studies are needed to explore the reverse regulation theory, particularly in understanding the potential correlation of FA values with CRP levels in the context of interferon-alpha activation (Rönnblom & Eloranta 2013).

Lower haemoglobin values in the study group correlated with lower FA, possibly linked to pSS activity. However, these associations did not remain significant after Benjamini–Hochberg correction for multiple testing. This pattern suggests that the observed effects may be modest and require validation in studies with greater statistical power. While mean haemoglobin levels in the study group were within normal limits (~ 12.36 g/dl, standard deviation ~ 1.49), ferritin values and the impact of iron on neuronal mitochondria might contribute (Seror et al. 2011). A limitation was the lack of iron metabolism assessment. Neutrophil reduction was observed in patients with lower FA values in both fornixes, crucial for memory functions affected in pSS (Margaretten 2017). The suspected immune-mediated small vessel vasculopathy in pSS involves CSF abnormalities, (Alexander et al. 1986; Alexander 1986; Sanders et al. 1987) complement activation, (Sanders et al. 1987)and brain histopathology (Sakakibara et al. 2004). In contrast to previous angiography results, our study found higher FA values when anti-SSA antibodies were present, suggesting potential protective roles or indirect effects on brain tissue, possibly involving complement and RF mechanisms.

Rheumatoid factor (RF) is a prevalent marker in pSS (40–60%), correlating with anti-SSA and anti-SSB antibodies and disease activity (Rönnblom & Eloranta 2013; Alexander et al. 1986; Alexander 1986). RF levels exhibited several significant correlations with lower FA values, and some of these relationships persisted after Benjamini–Hochberg correction, highlighting their robustness. In contrast, pathological RF was linked to reduced FA values in only one WM tract (superior cerebellar peduncle) at the uncorrected Pearson level, but this association did not survive false-discovery-rate adjustment, indicating that the effect may be modest in magnitude.

This region, crucial for cognitive and behavioural processing, may be linked to the fatigue experienced by pSS patients, a common and debilitating symptom (Rönnblom & Eloranta 2013). Cognitive impairment in pSS, often manifesting as brain fog or mild cognitive impairment, is controversially associated with degenerative dementia syndromes like Alzheimer’s disease (AD). Despite hypotheses suggesting pSS as an AD risk factor, studies lack consistent data, emphasizing the need for routine cognitive function testing, especially in patients with pathological RF levels (Rönnblom & Eloranta 2013; Alexander et al. 1986). A limitation was the absence of baseline psychological assessments, warranting further research in the subsequent projects.

In the analysed study group, the presence of cryoglobulins was associated with significantly lower FA values, supporting its role as one of the key markers of clinical activity in pSS. Although this association showed a consistent effect direction and reached significance in the uncorrected Pearson analysis, it did not remain significant after Benjamini–Hochberg adjustment, suggesting a subtle effect that may exceed the statistical power of the current sample. Cryoglobulins correlate with the risk of developing lymphoma, vasculitis and glomerular renal inflammation in the course of pSS, (Feist et al. 2009; Retamozo et al. 2019)as well as damage to brain structures, as demonstrated in this study. This marker should always be assessed when diagnosing pSS, and later during visits monitoring the activity of the disease. Especially because cryoglobulins are associated with early mortality in the course of small-vascular inflammation (Mohammed et al. 2009).

Of course, co-existing diseases can also affect the reduction in FA values. Hypertension (HT) was found in 5 patients with pSS. In this patient cohort, individuals with hypertension demonstrated lower FA values than those without, reaching significance in the initial Pearson comparison. Nevertheless, this effect did not withstand Benjamini–Hochberg correction for multiple testing. In each case, blood pressure values were normalized by the use of antihypertensive medications. Blood pressure should be assessed during monitoring visits in patients with pSS, taking into account the possibility of double adverse effects of both diseases on brain tissue.

In line with the underlying hypothesis, higher ESSDAI scores were associated with decreased FA values. While this correlation achieved nominal significance prior to adjustment, it did not remain significant following Benjamini–Hochberg correction, suggesting a modest effect that may require larger cohorts for confirmation. From a practical clinical perspective, a reduction in FA was shown in patients who had been diagnosed with lymphadenopathy at the time of diagnosis. In the study presented by Stergiou et al., the association of lymphadenopathy with the presence of anti-SSA or anti-SSB antibodies, lymphopenia, greater focus score, symptomatic spotting and occupation of the peripheral nervous system was demonstrated, (Stergiou et al. 2022) which supports the view that lymphadenopathy is associated with greater pSS activity. In patients with enlarged lymph nodes, the possibility of CNS involvement should be considered.

Another argument in favour of screening and monitoring of WM disruption in patients with pSS is the possibility of treatment (Ozgocmen 2008). It has been suggested that CNS disease can be treated in patients without lesions on conventional MRI. A study based on patients with MS showed that microstructural disruption of WM tracts detected on DTI precedes typical white matter hyperintensities (WMHs) in conventional MRI scans (Deppe et al. 2016). In pSS with progressive symptoms leading to neurological deficits, more aggressive therapy is required. The most commonly used drugs are oral or intravenous pulses of glucocorticosteroids and cyclophosphamide. There is also high hope for biological drugs, but their use is often questioned (Ozgocmen 2008). However, DTI could detect changes at the level of WM microstructure already in the preclinical stage and allow the identification of a group of patients with a higher probability of CNS involvement.

### Limitations

This study has several methodological limitations. The retrospective design inherently constrains control over data heterogeneity and introduces the possibility of selection bias. In addition, the single-center origin of the dataset may limit the external validity and reduce the robustness of model generalization across different acquisition settings or population structures. Furthermore, the imaging analysis did not encompass the Meckel’s cave compartment or additional trigeminal nerve segments, which restricts the anatomical completeness of the dataset and may prevent full characterization of microstructural alterations along the entirety of the nerve pathway. These factors should be considered when interpreting the present findings and when extending the methodology to broader clinical or computational contexts.

The main limitations concerning TractSeg pipline relate to its supervised nature - performance ultimately depends on the quality and anatomical definitions of the training tracts, and very small bundles like the fornix or anterior commissure remain more challenging. Although these constraints are shared with other state-of-the-art methods and do not outweigh the substantial practical advantages of TractSeg, (Wasserthal et al. 2018, 2019) particularly when compared with other DTI analysis approaches.

Although several white-matter tracts showed statistically significant FA reductions in pSS— most of them persisting after Benjamini–Hochberg correction—the overall pattern after adjusting for sex and age was heterogeneous, with many WM tracts demonstrating only uncorrected effects or no detectable differences. Likewise, correlations between rheumatologic markers and FA were mostly weak and rarely survived correction, indicating substantial tract-specific variability. This non-uniformity underscores the need for cautious interpretation, as many clinical–imaging associations demonstrated modest effect sizes and may be influenced by measurement noise, limited cohort size, and residual confounding. Future studies should therefore pursue larger, multi-center cohorts to improve statistical power and reduce susceptibility to sampling variability.

## Conclusions

1. DTI is a sensitive method to detect impaired integrity of cerebral WM tracts in patients with pSS. Multiple WM tracts showed a statistically significant reduction in FA values (*p* < 0.05).
2. The most significant reduction in FA values in pSS was found in the cerebellar peduncles. Other tracts with significant reduction in FA included: anterior thalamic radiations, a large group of thalamocortical, corticospinal tracts, uncinate fasciculi and others. A novel observation in our study is the disruption of microstructure of the cerebellar peduncles and both optic radiations in patients with pSS, which has not previously been reported in the available literature.
3. Numerous associations and correlations were found between neuroradiological parameters and rheumatological factors. There was a correlation of CNS white matter damage with low CRP values, with haemoglobin levels, and with the presence of cryoglobulins. No relationship was shown between the presence of pathological RF levels and CNS white matter damage, indicating a possible influence of the interleukin system. The whole observation of neuroradiological and rheumatological correlations requires further structured studies to confirm the above-mentioned hypotheses.
4. DTI of the brain is a non-contrast agent study that could be a very useful tool for screening and monitoring the severity of neuro-pSS. DTI could assist in the decision to start, monitor or change/intensify treatment.
5. The utility of automated methods for collecting measurements in DTI has been demonstrated. The usefulness of TractSeg’s algorithm allows for rapid and, above all, reproducible collection of qualitative and quantitative data. Most importantly, the method requires minimal intervention and provides automatic, reproducible segmentation and quantitative analysis of 72 white matter tracts based on brain DTI in approximately 4 min.

## Supplementary Information

Below is the link to the electronic supplementary material.

Supplementary File 1 (DOCX 446 KB)

## Data Availability

Due to patient confidentiality and institutional regulations, the imaging and clinical data used in this study cannot be made publicly available. Data access is restricted to the medical center and the participants themselves. The analysis code and full processing pipeline are available from the authors upon reasonable request, provided that the purpose for access is scientifically justified.

## Abbreviations

ACR: American College of Rheumatology
AD: Axial diffusivity / Alzheimer’s disease
AF: Arcuate fasciculus
ANA: Antinuclear antibodies
ATR: Anterior thalamic radiation
BRAVO: T1-weighted BRAVO MRI sequence
BH: Benjamini–Hochberg (correction)
CA: Anterior commissure
CC: Corpus callosum
CC1: Corpus callosum – rostrum
CC2: Corpus callosum – genu
CC3: Corpus callosum – rostral body
CC4: Corpus callosum – anterior body
CC5: Corpus callosum – posterior body
CC6: Corpus callosum – isthmus
CC7: Corpus callosum – splenium
CG: Cingulum
CI: Confidence interval
CNS: Central nervous system
CRP: C-reactive protein
CSF: Cerebrospinal fluid
CST: Corticospinal tract
DTI: Diffusion tensor imaging
EULAR: European Alliance of Associations for Rheumatology
ESSPRI: EULAR Sjögren’s Syndrome Patient Reported Index
ESSDAI: EULAR Sjögren’s Syndrome Disease Activity Index
FA: Fractional anisotropy
FCNN: Fully convolutional neural networks
FDR: False discovery rate
FLAIR: Fluid-attenuated inversion recovery
FOV: Field of view
FPT: Fronto-pontine tract
FTD: Frontotemporal dementia
FSPGR: Fast spoiled gradient echo
FX: Fornix
HB: Hemoglobin
HT: Hypertension
ICP: Inferior cerebellar peduncle
ICV: Intracranial volume
IFOF: Inferior fronto-occipital fasciculus
IL: Interleukin
ILF: Inferior longitudinal fasciculus
MCI: Mild cognitive impairment
MD: Mean diffusivity
MLF: Middle longitudinal fasciculus
MRI: Magnetic resonance imaging
MS: Multiple sclerosis
NMOSD: Neuromyelitis optica spectrum disorder
PET: Positron emission tomography
PNFA: Progressive nonfluent aphasia
POPT: Parieto-occipital pontine tract
pSS: primary Sjögren’s syndrome
RF: Rheumatoid factor
ROI: Region of interest
SCP: Superior cerebellar peduncle
SC: Structural connectivity
SD: Standard deviation / Semantic dementia
S: Systemic lupus erythematosus
SLF: Superior longitudinal fasciculus
SLF_I: Superior longitudinal fasciculus I
SLF_II: Superior longitudinal fasciculus II
SLF_III: Superior longitudinal fasciculus III
SMD: standardized mean differences; Cohen’s d
SNR: Signal-to-noise ratio
SPECT: Single Photon Emission Computed Tomography
ST_FO: Striato-fronto-orbital tract
ST_OCC: Striato-occipital tract
ST_PAR: Striato-parietal tract
ST_POSTC: Striato-postcentral tract
ST_PREC: Striato-precentral tract
ST_PREF: Striato-prefrontal tract
ST_PREM: Striato-premotor tract
STR: Superior thalamic radiation
TBSS: Tract-based spatial statistics
TOF: Time of flight
T_OCC: Thalamo-occipital tract
T_PAR: Thalamo-parietal tract
T_POSTC: Thalamo-postcentral tract
T_PREC: Thalamo-precentral tract
T_PREF: Thalamo-prefrontal tract
T_PREM: Thalamo-premotor tract
UF: Uncinate fasciculus
WM: White matter
WMH: White-matter hyperintensities
