# Supplementary material for: Application of Fully Convolutional Neural Networks in the Assessment of Cerebral White Matter Involvement in Primary Sjögren’s Syndrome
Source: Neuroinformatics. 2025 Dec 29;24(1):3. doi: 10.1007/s12021-025-09762-1 (PMC12748124; doi:10.1007/s12021-025-09762-1)
Supplement: Supplementary file 1 — (DOCX 446 KB) [file 12021_2025_9762_MOESM1_ESM.docx]

# Supplement

## Supplementary data 1.

TractSeg, introduced by J. Wasserthal in 2018, uses fully convolutional neural networks (FCNN) to directly segment WM tracts based on the fibre orientation distribution function (fODF). To overcome challenges in raw diffusion MRI scans, the data is transformed into fODF peaks, condensing information and reducing variance across studies. This approach extracts three dominant fibre directions per voxel, resulting in nine 3D images.^1^

The U-Net model (Figure 1) processes input images slice by slice (144x144), producing a 72-channel output indicating the probability of voxels belonging to WM bundles. In inference, segmentation happens three times for each orientation, and the slices are merged into a 144x144x144x216 output. This output is then fed into a second model, which optimally combines probability maps from the first model. ^2,3^


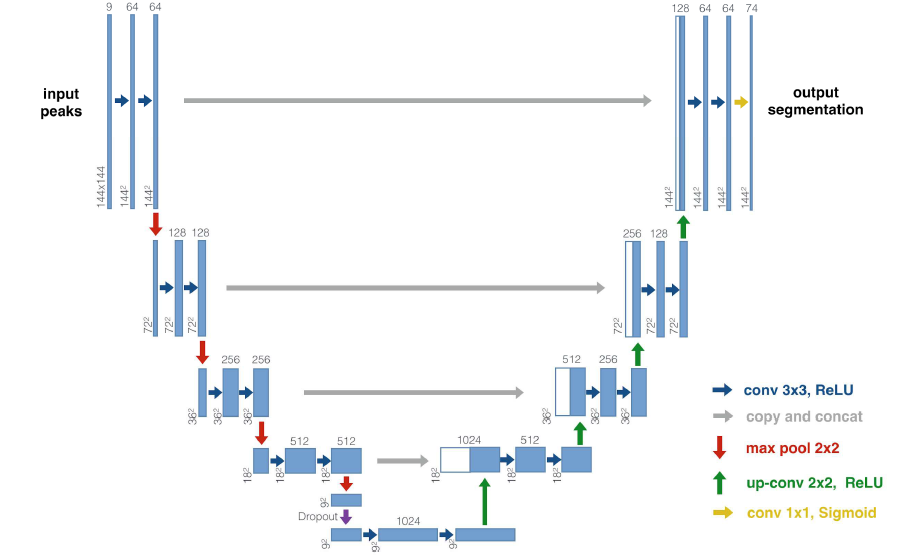


Figure 1 The main block used in the TractSeg113 architecture. The blue rectangles represent multi-channel feature maps. The white rectangles show the feature maps copied directly from a given encoder level to the same decoder level (bypassing the operations that are in the blocks below). The grey number at the top of each rectangle tells the number of channels. The map sizes, on the other hand, are given in the bottom left corner of each rectangle. Network operations are represented by different coloured arrows.

(Figure based on: Wasserthal J, Neher P, Maier-Hein KH. TractSeg - Fast and accurate WM tract segmentation. Neuroimage. 2018;183:239-253. doi:10.1016/j.neuroimage.2018.07.070.)

The algorithm was trained and tested on 105 patient imaging studies from the Human Connectome Project (HCP) with isotropic voxel size (1.25mm). Each study included 288 3D images, with 270 having gradient directions (b values: 1000, 2000, 3000 s/mm²) and 18 with b = 0 s/mm².^4^

Binary segmentations of the 72 main WM tracts which served as labels for training the artificial neural network, were generated by Wasserthal et al. in a semi-automatic manner described below in five steps (Figure 2).
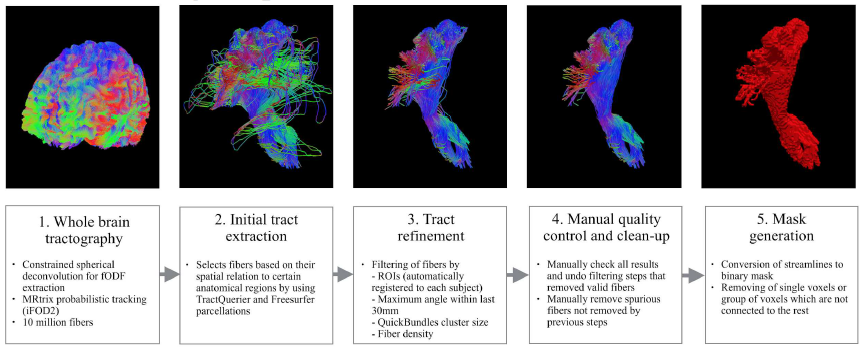


Figure 2 Methodology for semi-automatic segmentation of reference bundles.

(Figure based on: Wasserthal J, Neher P, Maier-Hein KH. TractSeg - Fast and accurate WM tract segmentation. Neuroimage. 2018;183:239-253. doi:10.1016/j.neuroimage.2018.07.070.)

## Supplementary data 2.

Fractional anisotropy and mean diffusivity are derived from diffusion magnetic resonance scans (dMRI) using a voxel-wise model. The diffusion tensor model, introduced by Basser et al. in 1994, enhances the efficacy of dMRI in characterizing WM microstructure through local diffusion properties, ^5^ representing the diffusion signal as:

where:

- g — three-dimensional unit vector indicating the measurement direction,
- b — strength and duration of the diffusion weighting gradient,
- S(g, b) — diffusion-weighted signal,
- S0 — signal acquired without diffusion weighting,
- D — a positively defined quadratic formulation containing six unknown parameters derived from the covariance matrix:

The covariance matrix represents diffusivity in three dimensions. Antipodal symmetry assumption equates elements on opposite sides of the diagonal (e.g., Dxy=Dyx), reducing the estimation to six independent parameters. Calculating the covariance matrix D, provides eigenvalues λ1, λ2, λ3.

Fractional anisotropy (FA) and mean diffusivity (MD) were computed from these eigenvalues using specific formulas.

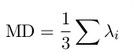


# References

1. Wasserthal J, Neher P, Maier-Hein KH. TractSeg - Fast and accurate white matter tract segmentation. *Neuroimage*. 2018;183:239-253. doi:10.1016/j.neuroimage.2018.07.070

2. Ronneberger O, Fischer P, Brox T. U-Net: Convolutional Networks for Biomedical Image Segmentation. Published online May 18, 2015. http://arxiv.org/abs/1505.04597

3. Dumoulin V, Visin F. A guide to convolution arithmetic for deep learning. Published online March 23, 2016. http://arxiv.org/abs/1603.07285

4. Glasser MF, Sotiropoulos SN, Wilson JA, et al. The minimal preprocessing pipelines for the Human Connectome Project. *Neuroimage*. 2013;80:105-124. doi:10.1016/j.neuroimage.2013.04.127

5. Basser PJ, Mattiello J, LeBihan D. MR diffusion tensor spectroscopy and imaging. *Biophys J*. 1994;66(1):259-267. doi:10.1016/S0006-3495(94)80775-1
